# (Acridine-κ*N*)(pyridine-2,6-dicarboxyl­ato-κ^3^ *O* ^2^,*N*,*O* ^6^)palladium(II)

**DOI:** 10.1107/S1600536812011385

**Published:** 2012-03-21

**Authors:** Kwang Ha

**Affiliations:** a School of Applied Chemical Engineering, Research Institute of Catalysis, Chonnam National University, Gwangju 500-757, Republic of Korea

## Abstract

In the title complex, [Pd(C_7_H_3_NO_4_)(C_13_H_9_N)], the Pd^II^ ion is four-coordinated in a distorted square-planar environment by one N and two O atoms from the tridentate pyridine-2,6-dicarboxyl­ate (dipic) anionic ligand and one N atom of the acridine (acr) ligand. The dipic and acr ligands are nearly planar [maximum deviation = 0.069 (3) Å in dipic and 0.091 (4) Å in acr] and the dihedral angle between their mean planes is 58.67 (7)°. The Pd—O bond lengths are nearly equal, but the Pd—N bond lengths are slightly different. There is a short C—H⋯O inter­action in the mol­ecule involving the two ligands. In the crystal, complex mol­ecules are linked through C—H⋯O inter­actions, forming a three-dimensional network. There are also a number of inter­molecular π–π inter­actions present, the shortest ring centroid–centroid distance being 3.622 (3) Å.

## Related literature

For the crystal structure of the related Pt^II^ complex [Pt(C_7_H_3_NO_4_)(C_13_H_9_N)], see: Ha (2011 ▶).
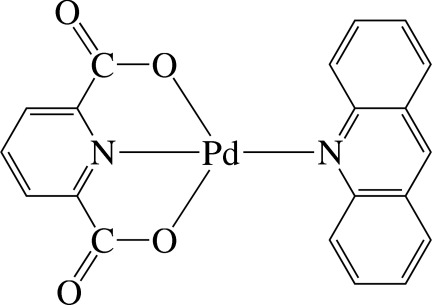


## Experimental

### 

#### Crystal data

[Pd(C_7_H_3_NO_4_)(C_13_H_9_N)]
*M* *_r_* = 450.72
Monoclinic, 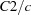

*a* = 25.299 (6) Å
*b* = 9.193 (2) Å
*c* = 13.917 (3) Å
β = 94.289 (5)°
*V* = 3227.8 (13) Å^3^
*Z* = 8
Mo *K*α radiation
μ = 1.18 mm^−1^
*T* = 200 K
0.19 × 0.18 × 0.14 mm

#### Data collection

Bruker SMART 1000 CCD diffractometer
Absorption correction: multi-scan (*SADABS*; Bruker, 2000 ▶) *T*_min_ = 0.754, *T*_max_ = 1.000
9414 measured reflections
3152 independent reflections
2263 reflections with *I* > 2σ(*I*)
*R*_int_ = 0.074

#### Refinement

*R*[*F* ^2^ > 2σ(*F* ^2^)] = 0.041
*wR*(*F* ^2^) = 0.100
*S* = 0.99
3152 reflections
244 parameters
H-atom parameters constrained
Δρ_max_ = 1.21 e Å^−3^
Δρ_min_ = −1.14 e Å^−3^

### 

Data collection: *SMART* (Bruker, 2000 ▶); cell refinement: *SAINT* (Bruker, 2000 ▶); data reduction: *SAINT*; program(s) used to solve structure: *SHELXS97* (Sheldrick, 2008 ▶); program(s) used to refine structure: *SHELXL97* (Sheldrick, 2008 ▶); molecular graphics: *ORTEP-3* (Farrugia, 1997 ▶) and *PLATON* (Spek, 2009 ▶); software used to prepare material for publication: *SHELXL97*.

## Supplementary Material

Crystal structure: contains datablock(s) global, I. DOI: 10.1107/S1600536812011385/su2392sup1.cif

Structure factors: contains datablock(s) I. DOI: 10.1107/S1600536812011385/su2392Isup2.hkl

Additional supplementary materials:  crystallographic information; 3D view; checkCIF report

## Figures and Tables

**Table:** 

Pd1—N1	1.923 (4)
Pd1—O1	2.036 (3)
Pd1—O3	2.037 (3)
Pd1—N2	2.063 (4)

**Table:** 

N1—Pd1—O1	81.25 (14)
N1—Pd1—O3	81.17 (14)

**Table 2:** Hydrogen-bond geometry (Å, °)

*D*—H⋯*A*	*D*—H	H⋯*A*	*D*⋯*A*	*D*—H⋯*A*
C19—H19⋯O3	0.95	2.48	3.196 (6)	132
C2—H2⋯O4^i^	0.95	2.26	3.193 (6)	166
C14—H14⋯O1^ii^	0.95	2.47	3.307 (6)	147
C18—H18⋯O4^iii^	0.95	2.47	3.285 (6)	144

Symmetry codes: (i) 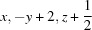; (ii) 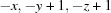; (iii) 
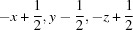
.

**Fig. 1.**
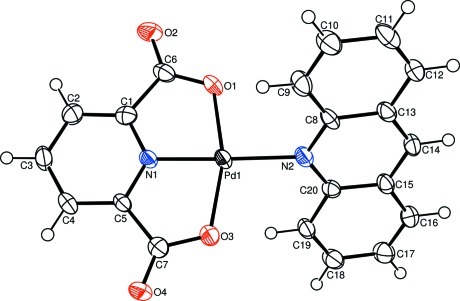
A view of the molecular structure of the title complex, with atom numbering. Displacement ellipsoids are drawn at the 50% probability level.

**Fig. 2.**
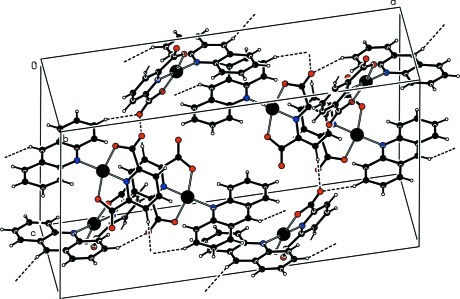
A view along the b axis of the crystal packing of the title complex. Intermolecular C—H···O interactions are drawn as dashed lines.

## supplementary crystallographic information

### Comment

The title complex is isomorphous with the previously reported analogous Pt^II^ complex [Pt(dipic)(acr)] (Ha, 2011).

In the title complex, the Pd^II^ ion is four-coordinated in a distorted square-planar environment by one N and two O atoms from the tridentate pyridine-2,6-dicarboxylate (dipic) anionic ligand and one N atom of the acridine (acr) ligand (Fig. 1). The main contribution to the distortion is the tight N—Pd—O chelate angles [N1—Pd1—O1 = 81.25 (14)° and N1—Pd1—O3 = 81.17 (14)°], which results in a non-linear *trans* arrangement of the O1—Pd1—O3 bond with 162.40 (12)°, whereas the N1—Pd1—N2 bond is almost linear, 178.40 (16)°. The Pd—O bond lengths are nearly equal [2.036 (3) Å and 2.037 (3) Å], but the Pd—N bond lengths are slightly different. The Pd1—N1(dipic) bond [1.923 (4) Å] is somewhat shorter than the Pd1—N2(acr) bond [2.063 (4) Å] (Table 1). The dipic and acr ligands are nearly planar [maximum deviation = 0.069 (3) Å in dipic and 0.091 (4) Å in acr] and the dihedral angle between the least-squares planes of the two ligands is 58.67 (7)°. In the molecule, there is a short C19—H19···O3 interaction involving the two ligands.

In the crystal, complex molecules are linked through C—H···O interactions, forming a three-dimensional network (Fig. 2 and Table 2). The crystal structure also displays numerous intermolecular π···π interactions between adjacent six-membered rings: Cg1···Cg1^i^ 3.822 (3) Å; Cg2···Cg2^ii^ 3.622 (3) Å; Cg2···Cg2^iii^ 3.854 (3) Å; Cg2···Cg3^iii^ 3.638 (3) Å; Cg3···Cg4^iii^ 3.986 (3) Å [Cg1, Cg2, Cg3 and Cg4 are the centroids of rings N1/C1-C5, N2/C8/C13-C15/C20, C8-C13 and C15-C20, respectively; symmetry codes: (i) x+1/2, -y+3/2, z+3/2; (ii) -x, y, -z+1/2; (iii) -x, -y+1, -z+1].

### Experimental

To a solution of acridine (0.0898 g, 0.501 mmol) in EtOH (20 ml) and MeOH (10 ml) were added pyridine-2,6-dicarboxylic acid (0.0838 g, 0.501 mmol) and Na_2_PdCl_4_ (0.1465 g, 0.498 mmol) and stirred for 3 h at room temperature. After addition of H_2_O (10 ml) to the reaction mixture, the formed precipitate was separated by filtration, washed with EtOH and ether, and dried at 333 K, to give a yellow powder (0.1546 g). Block-like yellow crystals, suitable for X-ray analysis, were obtained by slow evaporation of an acetone solution.

### Refinement

H atoms were positioned geometrically and allowed to ride on their respective parent atoms: C—H = 0.95 Å with *U*_iso_(H) = 1.2*U*_eq_(C). The highest peak (1.21 e Å^-3^) and the deepest hole (-1.14 e Å^-3^) in the difference Fourier map are located 1.38 Å and 0.99 Å, respectively, from the Pd1 atom.

### Crystal data

**Table:**

[Pd(C_7_H_3_NO_4_)(C_13_H_9_N)]	*F*(000) = 1792
*M**_r_* = 450.72	*D*_x_ = 1.855 Mg m^−^^3^
Monoclinic, *C*2/*c*	Mo *K*α radiation, λ = 0.71073 Å
Hall symbol: -C 2yc	Cell parameters from 3572 reflections
*a* = 25.299 (6) Å	θ = 2.4–25.8°
*b* = 9.193 (2) Å	µ = 1.18 mm^−^^1^
*c* = 13.917 (3) Å	*T* = 200 K
β = 94.289 (5)°	Block, yellow
*V* = 3227.8 (13) Å^3^	0.19 × 0.18 × 0.14 mm
*Z* = 8	

### Data collection

**Table:**

Bruker SMART 1000 CCD diffractometer	3152 independent reflections
Radiation source: fine-focus sealed tube	2263 reflections with *I* > 2σ(*I*)
Graphite monochromator	*R*_int_ = 0.074
φ and ω scans	θ_max_ = 26.0°, θ_min_ = 2.4°
Absorption correction: multi-scan (*SADABS*; Bruker, 2000)	*h* = −31→24
*T*_min_ = 0.754, *T*_max_ = 1.000	*k* = −11→11
9414 measured reflections	*l* = −16→17

### Refinement

**Table:**

Refinement on *F*^2^	Primary atom site location: structure-invariant direct methods
Least-squares matrix: full	Secondary atom site location: difference Fourier map
*R*[*F*^2^ > 2σ(*F*^2^)] = 0.041	Hydrogen site location: inferred from neighbouring sites
*wR*(*F*^2^) = 0.100	H-atom parameters constrained
*S* = 0.99	*w* = 1/[σ^2^(*F*_o_^2^) + (0.0299*P*)^2^] where *P* = (*F*_o_^2^ + 2*F*_c_^2^)/3
3152 reflections	(Δ/σ)_max_ = 0.001
244 parameters	Δρ_max_ = 1.21 e Å^−^^3^
0 restraints	Δρ_min_ = −1.14 e Å^−^^3^

### Special details

**Table:**

Geometry. All e.s.d.'s (except the e.s.d. in the dihedral angle between two l.s. planes) are estimated using the full covariance matrix. The cell e.s.d.'s are taken into account individually in the estimation of e.s.d.'s in distances, angles and torsion angles; correlations between e.s.d.'s in cell parameters are only used when they are defined by crystal symmetry. An approximate (isotropic) treatment of cell e.s.d.'s is used for estimating e.s.d.'s involving l.s. planes.
Refinement. Refinement of *F*^2^ against ALL reflections. The weighted *R*-factor *wR* and goodness of fit *S* are based on *F*^2^, conventional *R*-factors *R* are based on *F*, with *F* set to zero for negative *F*^2^. The threshold expression of *F*^2^ > σ(*F*^2^) is used only for calculating *R*-factors(gt) *etc*. and is not relevant to the choice of reflections for refinement. *R*-factors based on *F*^2^ are statistically about twice as large as those based on *F*, and *R*- factors based on ALL data will be even larger.

### Fractional atomic coordinates and isotropic or equivalent isotropic displacement parameters (Å^2^)

**Table:**

	*x*	*y*	*z*	*U*_iso_*/*U*_eq_	
Pd1	0.134139 (13)	0.71007 (4)	0.40295 (3)	0.02570 (14)	
O1	0.11671 (13)	0.7844 (4)	0.5347 (2)	0.0352 (8)	
O2	0.14722 (14)	0.9431 (4)	0.6479 (3)	0.0439 (9)	
O3	0.17122 (12)	0.6757 (3)	0.2800 (2)	0.0287 (8)	
O4	0.23893 (13)	0.7628 (3)	0.2027 (2)	0.0339 (8)	
N1	0.19313 (14)	0.8401 (4)	0.4277 (3)	0.0239 (8)	
C1	0.19562 (18)	0.9152 (5)	0.5092 (3)	0.0268 (11)	
C2	0.23707 (18)	1.0133 (5)	0.5271 (4)	0.0314 (11)	
H2	0.2407	1.0671	0.5855	0.038*	
C3	0.27301 (19)	1.0302 (5)	0.4574 (4)	0.0332 (12)	
H3	0.3012	1.0978	0.4678	0.040*	
C4	0.26854 (17)	0.9507 (5)	0.3732 (3)	0.0272 (11)	
H4	0.2935	0.9621	0.3261	0.033*	
C5	0.22685 (16)	0.8542 (5)	0.3591 (3)	0.0225 (10)	
C6	0.15056 (19)	0.8822 (5)	0.5717 (4)	0.0303 (11)	
C7	0.21303 (19)	0.7596 (5)	0.2724 (4)	0.0264 (11)	
N2	0.06979 (14)	0.5735 (4)	0.3794 (3)	0.0272 (9)	
C8	0.01981 (17)	0.6267 (5)	0.3831 (3)	0.0255 (10)	
C9	0.0109 (2)	0.7782 (5)	0.3909 (4)	0.0341 (12)	
H9	0.0401	0.8436	0.3943	0.041*	
C10	−0.0395 (2)	0.8306 (6)	0.3935 (4)	0.0379 (13)	
H10	−0.0448	0.9325	0.3993	0.046*	
C11	−0.08419 (19)	0.7374 (6)	0.3878 (4)	0.0386 (13)	
H11	−0.1189	0.7767	0.3878	0.046*	
C12	−0.07704 (18)	0.5922 (6)	0.3823 (4)	0.0327 (12)	
H12	−0.1069	0.5294	0.3802	0.039*	
C13	−0.02531 (18)	0.5317 (5)	0.3796 (3)	0.0273 (11)	
C14	−0.01654 (18)	0.3828 (5)	0.3726 (3)	0.0305 (11)	
H14	−0.0456	0.3177	0.3735	0.037*	
C15	0.03403 (18)	0.3279 (5)	0.3645 (3)	0.0270 (11)	
C16	0.0441 (2)	0.1783 (5)	0.3529 (4)	0.0343 (12)	
H16	0.0157	0.1109	0.3531	0.041*	
C17	0.0936 (2)	0.1295 (5)	0.3414 (4)	0.0361 (12)	
H17	0.0995	0.0284	0.3330	0.043*	
C18	0.1361 (2)	0.2263 (5)	0.3419 (4)	0.0349 (12)	
H18	0.1705	0.1906	0.3325	0.042*	
C19	0.12879 (18)	0.3722 (5)	0.3557 (3)	0.0283 (11)	
H19	0.1583	0.4364	0.3570	0.034*	
C20	0.07736 (17)	0.4280 (5)	0.3680 (3)	0.0248 (10)	

### Atomic displacement parameters (Å^2^)

**Table:**

	*U*^11^	*U*^22^	*U*^33^	*U*^12^	*U*^13^	*U*^23^
Pd1	0.0201 (2)	0.0276 (2)	0.0302 (2)	−0.00417 (15)	0.00736 (14)	0.00110 (17)
O1	0.0278 (19)	0.041 (2)	0.039 (2)	−0.0089 (15)	0.0170 (15)	−0.0026 (17)
O2	0.053 (2)	0.047 (2)	0.035 (2)	−0.0052 (18)	0.0240 (18)	−0.0064 (18)
O3	0.0262 (18)	0.0322 (19)	0.0281 (19)	−0.0041 (14)	0.0041 (14)	−0.0009 (14)
O4	0.037 (2)	0.038 (2)	0.029 (2)	−0.0028 (15)	0.0153 (16)	−0.0019 (15)
N1	0.023 (2)	0.024 (2)	0.025 (2)	−0.0022 (16)	0.0078 (16)	0.0022 (17)
C1	0.031 (3)	0.023 (2)	0.027 (3)	0.005 (2)	0.005 (2)	0.003 (2)
C2	0.033 (3)	0.034 (3)	0.027 (3)	−0.003 (2)	0.001 (2)	−0.003 (2)
C3	0.030 (3)	0.034 (3)	0.036 (3)	−0.014 (2)	0.004 (2)	0.001 (2)
C4	0.021 (3)	0.030 (3)	0.032 (3)	−0.0014 (19)	0.007 (2)	0.008 (2)
C5	0.018 (2)	0.024 (2)	0.027 (3)	0.0002 (18)	0.0066 (19)	0.003 (2)
C6	0.030 (3)	0.030 (3)	0.032 (3)	0.003 (2)	0.013 (2)	0.005 (2)
C7	0.027 (3)	0.023 (3)	0.029 (3)	0.002 (2)	0.004 (2)	0.004 (2)
N2	0.022 (2)	0.032 (2)	0.028 (2)	−0.0041 (17)	0.0055 (17)	0.0018 (17)
C8	0.018 (2)	0.037 (3)	0.022 (3)	−0.003 (2)	0.0056 (18)	0.006 (2)
C9	0.031 (3)	0.028 (3)	0.044 (3)	−0.004 (2)	0.009 (2)	0.010 (2)
C10	0.031 (3)	0.035 (3)	0.049 (4)	0.004 (2)	0.011 (2)	0.006 (2)
C11	0.019 (3)	0.057 (4)	0.040 (3)	0.003 (2)	0.004 (2)	0.004 (3)
C12	0.019 (3)	0.041 (3)	0.038 (3)	−0.003 (2)	0.004 (2)	−0.006 (2)
C13	0.023 (3)	0.038 (3)	0.022 (3)	−0.005 (2)	0.0060 (19)	0.002 (2)
C14	0.025 (3)	0.034 (3)	0.033 (3)	−0.011 (2)	0.005 (2)	−0.001 (2)
C15	0.023 (3)	0.037 (3)	0.021 (3)	−0.008 (2)	0.0039 (19)	0.002 (2)
C16	0.033 (3)	0.031 (3)	0.039 (3)	−0.011 (2)	0.010 (2)	−0.002 (2)
C17	0.040 (3)	0.028 (3)	0.042 (3)	−0.003 (2)	0.013 (2)	0.002 (2)
C18	0.031 (3)	0.039 (3)	0.037 (3)	0.000 (2)	0.011 (2)	0.001 (2)
C19	0.023 (3)	0.031 (3)	0.031 (3)	−0.006 (2)	0.006 (2)	0.002 (2)
C20	0.025 (3)	0.030 (3)	0.020 (2)	−0.004 (2)	0.0053 (19)	0.0005 (19)

### Geometric parameters (Å, º)

**Table:**

Pd1—N1	1.923 (4)	C8—C13	1.435 (6)
Pd1—O1	2.036 (3)	C9—C10	1.367 (7)
Pd1—O3	2.037 (3)	C9—H9	0.9500
Pd1—N2	2.063 (4)	C10—C11	1.416 (7)
O1—C6	1.319 (6)	C10—H10	0.9500
O2—C6	1.208 (6)	C11—C12	1.350 (7)
O3—C7	1.320 (6)	C11—H11	0.9500
O4—C7	1.211 (6)	C12—C13	1.425 (6)
N1—C1	1.325 (6)	C12—H12	0.9500
N1—C5	1.333 (6)	C13—C14	1.391 (6)
C1—C2	1.391 (6)	C14—C15	1.388 (6)
C1—C6	1.515 (6)	C14—H14	0.9500
C2—C3	1.388 (7)	C15—C16	1.410 (7)
C2—H2	0.9500	C15—C20	1.429 (6)
C3—C4	1.378 (7)	C16—C17	1.351 (7)
C3—H3	0.9500	C16—H16	0.9500
C4—C5	1.381 (6)	C17—C18	1.395 (7)
C4—H4	0.9500	C17—H17	0.9500
C5—C7	1.507 (7)	C18—C19	1.369 (7)
N2—C8	1.360 (5)	C18—H18	0.9500
N2—C20	1.362 (6)	C19—C20	1.421 (6)
C8—C9	1.417 (6)	C19—H19	0.9500
			
N1—Pd1—O1	81.25 (14)	C9—C8—C13	118.1 (4)
N1—Pd1—O3	81.17 (14)	C10—C9—C8	120.2 (4)
O1—Pd1—O3	162.40 (12)	C10—C9—H9	119.9
N1—Pd1—N2	178.40 (16)	C8—C9—H9	119.9
O1—Pd1—N2	97.22 (14)	C9—C10—C11	121.9 (5)
O3—Pd1—N2	100.37 (14)	C9—C10—H10	119.1
C6—O1—Pd1	113.7 (3)	C11—C10—H10	119.1
C7—O3—Pd1	113.5 (3)	C12—C11—C10	119.5 (5)
C1—N1—C5	124.7 (4)	C12—C11—H11	120.3
C1—N1—Pd1	117.6 (3)	C10—C11—H11	120.3
C5—N1—Pd1	117.5 (3)	C11—C12—C13	121.0 (4)
N1—C1—C2	118.6 (4)	C11—C12—H12	119.5
N1—C1—C6	113.4 (4)	C13—C12—H12	119.5
C2—C1—C6	128.0 (4)	C14—C13—C12	122.5 (4)
C3—C2—C1	118.1 (5)	C14—C13—C8	118.1 (4)
C3—C2—H2	120.9	C12—C13—C8	119.4 (4)
C1—C2—H2	120.9	C15—C14—C13	121.1 (4)
C4—C3—C2	121.3 (4)	C15—C14—H14	119.4
C4—C3—H3	119.4	C13—C14—H14	119.4
C2—C3—H3	119.4	C14—C15—C16	122.7 (4)
C3—C4—C5	118.3 (4)	C14—C15—C20	118.2 (4)
C3—C4—H4	120.8	C16—C15—C20	119.2 (4)
C5—C4—H4	120.8	C17—C16—C15	121.0 (4)
N1—C5—C4	118.9 (4)	C17—C16—H16	119.5
N1—C5—C7	113.2 (4)	C15—C16—H16	119.5
C4—C5—C7	127.9 (4)	C16—C17—C18	120.5 (5)
O2—C6—O1	124.9 (4)	C16—C17—H17	119.7
O2—C6—C1	121.1 (4)	C18—C17—H17	119.7
O1—C6—C1	114.0 (4)	C19—C18—C17	120.9 (5)
O4—C7—O3	124.3 (4)	C19—C18—H18	119.5
O4—C7—C5	121.3 (4)	C17—C18—H18	119.5
O3—C7—C5	114.4 (4)	C18—C19—C20	120.3 (4)
C8—N2—C20	119.8 (4)	C18—C19—H19	119.9
C8—N2—Pd1	120.0 (3)	C20—C19—H19	119.9
C20—N2—Pd1	120.0 (3)	N2—C20—C19	120.4 (4)
N2—C8—C9	120.7 (4)	N2—C20—C15	121.5 (4)
N2—C8—C13	121.3 (4)	C19—C20—C15	118.0 (4)
			
N1—Pd1—O1—C6	1.6 (3)	O3—Pd1—N2—C8	−124.7 (3)
O3—Pd1—O1—C6	4.0 (6)	O1—Pd1—N2—C20	−118.8 (3)
N2—Pd1—O1—C6	−178.0 (3)	O3—Pd1—N2—C20	60.6 (4)
N1—Pd1—O3—C7	−4.1 (3)	C20—N2—C8—C9	−177.3 (4)
O1—Pd1—O3—C7	−6.6 (6)	Pd1—N2—C8—C9	8.0 (6)
N2—Pd1—O3—C7	175.4 (3)	C20—N2—C8—C13	2.9 (6)
O1—Pd1—N1—C1	−1.5 (3)	Pd1—N2—C8—C13	−171.8 (3)
O3—Pd1—N1—C1	179.3 (3)	N2—C8—C9—C10	179.3 (5)
O1—Pd1—N1—C5	−177.7 (3)	C13—C8—C9—C10	−0.9 (7)
O3—Pd1—N1—C5	3.0 (3)	C8—C9—C10—C11	−0.5 (8)
C5—N1—C1—C2	−2.1 (7)	C9—C10—C11—C12	1.9 (8)
Pd1—N1—C1—C2	−178.0 (3)	C10—C11—C12—C13	−1.7 (8)
C5—N1—C1—C6	177.1 (4)	C11—C12—C13—C14	−179.1 (5)
Pd1—N1—C1—C6	1.2 (5)	C11—C12—C13—C8	0.2 (7)
N1—C1—C2—C3	1.7 (7)	N2—C8—C13—C14	0.2 (6)
C6—C1—C2—C3	−177.4 (4)	C9—C8—C13—C14	−179.5 (4)
C1—C2—C3—C4	−1.1 (7)	N2—C8—C13—C12	−179.1 (4)
C2—C3—C4—C5	0.7 (7)	C9—C8—C13—C12	1.1 (6)
C1—N1—C5—C4	1.8 (7)	C12—C13—C14—C15	176.2 (4)
Pd1—N1—C5—C4	177.7 (3)	C8—C13—C14—C15	−3.2 (7)
C1—N1—C5—C7	−177.5 (4)	C13—C14—C15—C16	−177.0 (4)
Pd1—N1—C5—C7	−1.5 (5)	C13—C14—C15—C20	2.9 (7)
C3—C4—C5—N1	−1.0 (7)	C14—C15—C16—C17	177.4 (5)
C3—C4—C5—C7	178.1 (4)	C20—C15—C16—C17	−2.5 (7)
Pd1—O1—C6—O2	178.3 (4)	C15—C16—C17—C18	0.7 (8)
Pd1—O1—C6—C1	−1.4 (5)	C16—C17—C18—C19	1.2 (8)
N1—C1—C6—O2	−179.4 (4)	C17—C18—C19—C20	−1.2 (7)
C2—C1—C6—O2	−0.3 (8)	C8—N2—C20—C19	174.6 (4)
N1—C1—C6—O1	0.2 (6)	Pd1—N2—C20—C19	−10.7 (6)
C2—C1—C6—O1	179.3 (4)	C8—N2—C20—C15	−3.2 (6)
Pd1—O3—C7—O4	−175.6 (4)	Pd1—N2—C20—C15	171.5 (3)
Pd1—O3—C7—C5	4.4 (5)	C18—C19—C20—N2	−178.4 (4)
N1—C5—C7—O4	177.9 (4)	C18—C19—C20—C15	−0.6 (7)
C4—C5—C7—O4	−1.2 (7)	C14—C15—C20—N2	0.3 (7)
N1—C5—C7—O3	−2.1 (6)	C16—C15—C20—N2	−179.8 (4)
C4—C5—C7—O3	178.8 (4)	C14—C15—C20—C19	−177.5 (4)
O1—Pd1—N2—C8	55.9 (3)	C16—C15—C20—C19	2.4 (6)

### Hydrogen-bond geometry (Å, º)

**Table:**

*D*—H···*A*	*D*—H	H···*A*	*D*···*A*	*D*—H···*A*
C19—H19···O3	0.95	2.48	3.196 (6)	132
C2—H2···O4^i^	0.95	2.26	3.193 (6)	166
C14—H14···O1^ii^	0.95	2.47	3.307 (6)	147
C18—H18···O4^iii^	0.95	2.47	3.285 (6)	144

Symmetry codes: (i) *x*, −*y*+2, *z*+1/2; (ii) −*x*, −*y*+1, −*z*+1; (iii) −*x*+1/2, *y*−1/2, −*z*+1/2.
